# A Clinical Prediction Scoring System for Cephalosporin Resistance Among Enteric Uropathogens: Indications and Practicality

**DOI:** 10.1093/ofid/ofz238

**Published:** 2019-06-13

**Authors:** Maroun M Sfeir

**Affiliations:** 1 Department of Pathology, Massachusetts General Hospital, Boston, Massachusetts; 2 Department of Pathology, Harvard Medical School, Boston, Massachusetts

Dear Editor,

We read with great pleasure the recently published study by Weinstein et al. [1]. This case–control study included a total of 302 patients seen in 2 emergency departments or in affiliated clinics in Philadelphia, Pennsylvania, between 2010 and 2013 with positive urine culture for *Enterobacterales*, and it matched cases who had extended-spectrum cephalosporin (EC)–resistant enteric uropathogens (EUs) 1:1 with controls with EC-susceptible EUs to predict a scoring system tool for EC resistance [1]. After multivariate analysis, a model was created that included 5 covariates: (1) a history of malignancy; (2) a history of diabetes; (3) recent skilled nursing facility or hospital stay; (4) recent trimethoprim-sulfamethoxazole exposure; and (5) pyelonephritis at the time of presentation. A score of ≥2 of the covariates is considered predictive of EC resistance with a sensitivity and specificity of 80% and 54%, respectively [1].

This clinical scoring system is highly useful for empiric antibiotic treatment for urinary tract infections (UTIs), especially the complicated ones, which often require treatment based on in vitro antibiotic susceptibility testing. The Infectious Diseases Society of America (IDSA) and the European Society for Microbiology and Infectious Diseases (ESCMID) guidelines for UTI emphasize the importance of empiric treatment of uncomplicated UTI without a urine culture and susceptibility testing. Those guidelines recommend either a single dose of oral fosfomycin, 5 days of oral nitrofurantoin, 3–7 days of pivmecillinam (which is only available in Europe and is not licensed for use in North America), 3–7 days of oral β-lactam (eg, amoxicillin-clavulanate, cefopodoxime, cefixime, etc.), or 3 days of oral trimethoprim-sulfamethoxazole (only if the local resistance rates of uropathogens are known and do not exceed 20%) [2]. Due to minimal resistance and low propensity for ecologic adverse effects, the former 3 antibiotics retain to a high extent activity against EC-resistant *Enterobacterales* [2]. For instance, a systematic review of 17 studies showed that fosfomycin was active against 96.8% (1604 of 1657) of EC-resistant *Escherichia coli* isolates and 81.3% (608 of 748) of EC-resistant *Klebsiella pneumoniae* [3]. Correspondingly, Tasbakan et al. showed that the relapse rate of EC-resistant *E. coli* after treatment with oral nitrofurantoin was 3.2% (1/31) at 4 weeks after finishing therapy [4]. Weinstein et al. did lump outpatients with patients seen in the emergency departments together and did not evaluate the complicated and uncomplicated UTIs separately. To that end, a subgroup analysis of the inpatients and/or patients with complicated UTIs, who often require hospital admission, would have been of great value to predict CE resistance and choose the appropriate empiric antibiotic treatment while awaiting susceptibility testing.

 Additionally, urine cultures are regularly ordered in healthy outpatient women with urinary symptoms even though they are often of limited value given the delay in obtaining results, the cost and inconvenience to the patient, and the high predictive value of symptoms alone in diagnosing acute cystitis [2]. In some circumstances where fosfomycin or nitrofurantoin cannot be used due to cost, allergy, or previous known resistance, a beta-lactam would be the alternative. In that situation, relying on a scoring tool to predict antimicrobial resistance and guide clinicians in choosing the appropriate antibiotic therapy for uncomplicated UTI without urine culture and susceptibility testing deserves praise.

The clinical tool developed by Weinstein et al. to predict EC resistance included recent trimethoprim-sulfamethoxazole exposure as unexpectedly the only antibiotic found to be significantly associated with EC resistance after multivariate analysis [1]. Although the bivariate analysis showed a significant correlation between EC exposure and EC resistance, after multivariate analysis, that correlation was not significant [1]. That is discordant with previous studies, which have demonstrated an association between EC and/or quinolones exposure but not trimethoprim-sulfamethoxazole and the production of extended-spectrum β-lactamase [5–7]. Moreover, the 2 prior studies that have created similar clinical prediction tools to predict EC resistance among uropathogens in inpatients only showed a significant correlation with previous antibiotic(s) exposure and EC resistance [8, 9]. However, neither of the 2 studies evaluated which class of antibiotics was the most implicated in EC resistance [8, 9]. Thus, larger studies are needed to validate and expand the current findings.

 In conclusion, the clinical prediction tool for EC resistance developed by Weinstein et al. could be most useful in the setting of empiric treatment choice for complicated UTI, but may be used less often in the case of uncomplicated UTI, where adherence to the IDSA and ESCMID UTI treatment guidelines would be the gold standard for empiric antimicrobial treatment [10]. One could also hope the advance in rapid microbiological testing could achieve rapid in vitro susceptibility testing that could provide antibiotic susceptibility results within minutes on urine specimens. Then, there would be no concern about relying on less sensitive and specific clinical tools to predict antimicrobial resistance.
